# Potato glycoside alkaloids exhibit antifungal activity by regulating the tricarboxylic acid cycle pathway of *Fusarium solani*

**DOI:** 10.3389/fmicb.2024.1390269

**Published:** 2024-04-15

**Authors:** Chongqing Zhang, Wei Chen, Bin Wang, Yupeng Wang, Nan Li, Ruiyun Li, Yuke Yan, Yuyan Sun, Jing He

**Affiliations:** ^1^College of Forestry, Gansu Agricultural University, Lanzhou, China; ^2^Wolfberry Harmless Cultivation Engineering Research Center of Gansu Province, Lanzhou, China

**Keywords:** potato glycoside alkaloids, *Fusarium solani*, tricarboxylic acid cycle, mitochondrion structure, gene expression

## Abstract

*Fusarium solani* is a pathogenic fungus that causes significant harm, leading to crop yield reduction, fruit quality reduction, postharvest decay, and other diseases. This study used potato glycoside alkaloids (PGA) as inhibitors to investigate their effects on the mitochondrial structure and tricarboxylic acid (TCA) cycle pathway of *F. solani*. The results showed that PGA could inhibit the colony growth of *F. solani* (54.49%), resulting in the disappearance of the mitochondrial membrane and the loss of contents. PGA significantly decreased the activities of aconitase (ACO), isocitrate dehydrogenase (IDH), α-ketoglutarate dehydrogenase (α-KGDH), succinate dehydrogenase (SDH), fumarase (FH), malate dehydrogenase (MDH), succinyl-CoA synthetase (SCS), and increased the activity of citrate synthase (CS) in *F. solani*. After PGA treatment, the contents of acetyl coenzyme A (CoA), citric acid (CA), malic acid (L-MA), and α-ketoglutaric acid (α-KG) in *F. solani* were significantly decreased. The contents of isocitric acid (ICA), succinyl coenzyme A (S-CoA), succinic acid (SA), fumaric acid (FA), and oxaloacetic acid (OA) were significantly increased. Transcriptomic analysis showed that PGA could significantly affect the expression levels of 19 genes related to TCA cycle in *F. solani*. RT-qPCR results showed that the expression levels of ACO, IDH, α-KGDH, and MDH-related genes were significantly down-regulated, and the expression levels of SDH and FH-related genes were significantly up-regulated, which was consistent with the results of transcriptomics. In summary, PGA can achieve antifungal effects by reducing the tricarboxylic acid cycle’s flow and regulating key genes’ expression levels. This study reveals the antifungal mechanism of PGA from the perspective of TCA cycle, and provides a theoretical basis for the development and application of PGA as a biopesticide.

## 1 Introduction

*Fusarium solani* is one of the most serious soil-borne pathogens in the world, which seriously affects the yield and quality of agricultural and forestry products. Its conidia can survive in soil for a long time. When the germination conditions are suitable, they can infect the vascular bundle tissues of various food crops, economic crops, medicinal plants, and ornamental plants through minor wounds, causing a series of plant rot diseases such as root rot, stem rot, ear rot, stem base rot and flower rot (Leslie and Summerell, 2006; Parikh et al., 2018; Mulero-Aparicio et al., 2019; Rampersad, 2020). Currently, the control of plant diseases caused by *F. solani* mainly depends on chemical fungicides, such as carbendazim (Beneduzi et al., 2012; Xu et al., 2018). However, the extensive use of chemical agent can lead to problems such as pesticide residues and drug resistance of pathogenic fungi, seriously endangering the soil environment and potential risks to human health (Fan et al., 2021). So it is urgent to finding an environmentally friendly green control method to control plant diseases caused by *F. solani*.

Potato glycoside alkaloids (PGA), also known as solanine, are sugar derivatives of an odor steroidal alkaloid found in potato plants and tubers (Schieber and Saldaña, 2009; Sanchez-Maldonado et al., 2014). More than 95% of PGA is α-solanine and α-chaconine (Friedman, 2006). PGA has strong biological activity, such as antifungal and pest resistance (Dahlin et al., 2017; Pillai and Dandurand, 2021). It has been reported that PGA can inhibit the growth and development of *Botrytis cinerea* (Sun et al., 2014), *Pectobacterium carotovorum* (Rocha et al., 2015), *Alternaria alternata* and *Pyrenophora tritici-repentis* (Sanchez-Maldonado et al., 2016), *Phytophthora infestans* (Dahlin et al., 2017). PGA could inhibit the spore germination of *Curvularia trifolii* (Xu et al., 2023), reduce the virulence of *Pectobacterium brasiliense* (Joshi et al., 2021), inhibit the active oxygen metabolism process of *F. sulphureum* to exert antifungal effect (Li et al., 2023).

Mitochondria are double-membrane-coated organelles that supply energy to cells by generating ATP through the tricarboxylic acid (TCA) cycle and oxidative phosphorylation (Fernie et al., 2004). TCA cycle provides substrates for oxidative phosphorylation and plays an important role in ATP synthesis (Kader and Saltveit, 2002). Any mitochondrial dysfunction can affect the growth of pathogenic fungi (Castelli et al., 2005). Therefore, mitochondria are often used as potential targets for developing antifungal drugs, some plant-derived extracts have shown this ability. 3-phenyllactic acid inhibited the growth of *Rhizopus oryzae* by disrupting the TCA cycle and affecting their energy metabolism (Fan et al., 2022). O-vanillin exerted antifungal effects by affecting the mitochondrial structure and TCA cycle of *Aspergillus flavus* (Li et al., 2022). Citral can inhibit the function of mitochondria of *Penicillium digitatum* to exert antifungal effect (Zheng et al., 2015). Eugenol and citral killed *A. niger* by tricarboxylic acid cycle (Ju et al., 2023).

Previous studies have shown that PGA can destroy the structure of mycelium by interfering with substance metabolism, inhibiting respiration and reducing ATP production (He et al., 2021; Ding et al., 2023; Zhang et al., 2023). However, the effect of PGA on the TCA cycle of *F. solani* has not been reported, and its mechanism still needs to be clarified.

**Figure 1:**
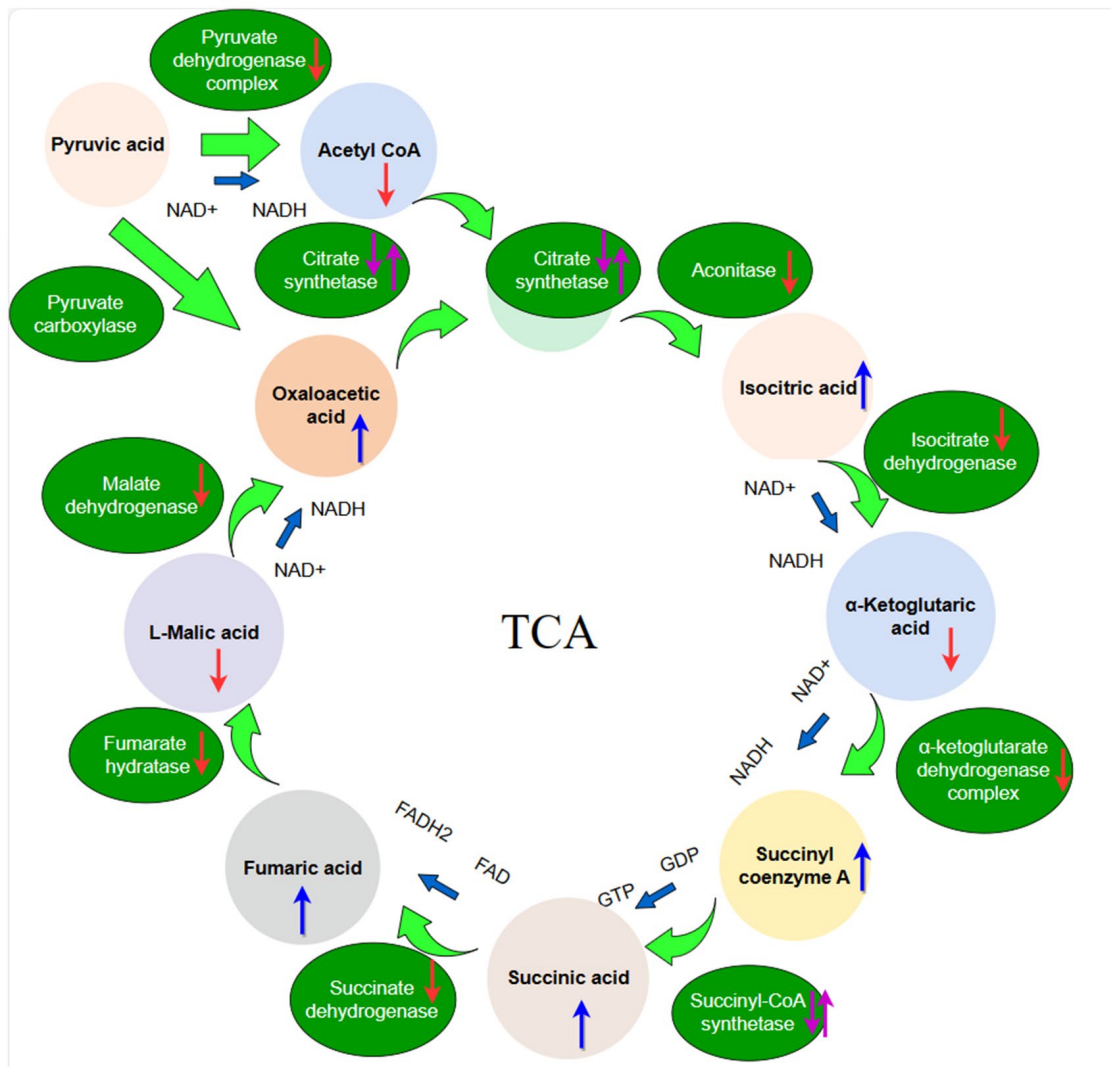
The schematic diagram of the TCA cycle. The red arrow indicates organic acid content or enzyme activities decreased, the purple arrow indicates organic acid content or enzyme activities first decreased and then increased, the bule arrow indicates organic acid content or enzyme activities increased.

Therefore, this study observed the effect of plant-derived extract PGA on mitochondrial function of *F. solani*. To reveal the potential antifungal mechanism of PGA inhibiting the growth of *F. solani* from the perspective of the TCA cycle, the changes of TCA-related enzyme activity and intermediate product content were determined, and the effect of PGA treatment on the expression of *F. solani* gene was further analyzed by transcriptome technology (Figure 1). This study aims to reveal the potential inhibitory mechanism of PGA in inhibiting the growth of *F. solani* from the perspective of the TCA cycle, and also to provide a theoretical basis for the development and utilization of antifungal active substances.

## 2 Materials and methods

### 2.1 Materials

The test strain, *F. solani*, was isolated from the diseased plants of wolfberry root rot and stored in the Forest Protection Laboratory of Forestry College of Gansu Agricultural University after pathogenicity determination. Before use, it was activated on a PDA plate and stored at 4°C for later use.

PGA was extracted and identified with reference to the team’s previous study (Zhang et al., 2023).

### 2.2 Antifungal effect of PGA

The agar plate diffusion method determined the antifungal activity of PGA (Huang et al., 2012). Four small holes (5 mm) 2.5 cm away from the center of the plate were drilled on the potato dextrose agar (PDA) plate using a sterile puncher. The fungal cake (5 mm in diameter) of *F. solani* was inoculated in the center of PDA plate. and 20 μL of PGA (88.10 mg·mL^−1^) was added to 2 small holes, The other two holes were added with the same amount of sterile distilled water as the control. All plates were cultured at 25°C for 5 days, and the clear inhibition zone around the pores was measured.

### 2.3 Observations of mitochondrial ultrastructure

We found that the EC_50_ value of PGA inhibiting *F. solani* was 4.4327 mg·mL^−1^ (Ding et al., 2023). Therefore, the concentration corresponding to EC_50_ was used as the treatment concentration of PGA in this experiment. 0.5 g fresh mycelium was inoculated in the potato dextrose broth (PDB) medium containing PGA (150 mL conical flask containing 30 mL of PDB medium) and oscillatory cultured (25°C, 160 rpm) for 9 h. And sterile water was set as the control. After 9 h, the mycelia were collected, centrifuged at 10,000 g for 10 min, and washed with sterile water 3–4 times.

The mitochondria of *F. solani* were extracted by differential centrifugation (Galina et al., 2004; Zhang et al., 2023). The mitochondria were fixed with 2.5% glutaraldehyde for 2 h, adjusted to pH 7.4 with 0.1 mol·L^−1^ phosphate buffer, and then fixed with 1% osmic acid at room temperature for 2 h. The samples were dehydrated in different ethanol concentrations (30%, 50%, 70%, 80%, 95%, and 100%) and immersed in epoxy resin and SPI-812 embedding agent, respectively. The ultra-thin sections obtained by the Leica UC7 ultra-thin slicing machine were stained with 2% uranyl acetate saturated alcohol solution in the dark and then stained with 2.6% lead citrate solution in the dark. Transmission electron microscopy (Hitachi HT7800, Japan) was used to observe and record images at 80 kV.

### 2.4 Determination of TCA-related enzyme activity

0.5 g fresh mycelium was inoculated into PDB culture medium (containing 4.432 7 mg·mL^−1^ PGA) and oscillatory cultured (25°C, 160 rpm) for 36 h. The mycelia were collected at 0, 3, 6, 9, 12, 24, and 36 h.

The activity of citrate synthase (CS) was determined by the methods of Jenner et al. (2001) and Li et al. (2022). One unit of enzyme activity was defined as the production of 1 nmol TNB per minute per gram of tissue in the reaction system at 25°C (U·g^−1^ FW). The activity of succinyl-CoA synthetase (SCS) was determined by the method of Vichaiya et al. (2020). The production of 1 μmol succinyl hydroxamic acid per minute per gram of tissue in the reaction system at 25°C was defined as an enzyme activity unit (U·g^−1^ FW). The activities of pyruvate dehydrogenase (PDH), aconitase (ACO), isocitrate dehydrogenase (IDH), α-ketoglutarate dehydrogenase (α-KGDH), succinate dehydrogenase (SDH), fumarase (FH) and malate dehydrogenase (MDH) were determined using the corresponding ELISA kits. The corresponding product numbers of the kits were G0836F, G0872F, G0833F, G0840F, G0856, G0869F, and G0820F. All of the above kits are from Suzhou Grace Biotechnology Co., Ltd.

### 2.5 Determination of TCA organic acid content

Acetyl-CoA content (CoA) was determined by the ELISA kit (JLC57299, Shanghai Jingkang Bioengineering Co., Ltd.). The contents of citric acid (CA) and L-malic acid (L-MA) were determined by kits. The kit numbers were G0864F and G0862F, respectively, provided by Suzhou Grace Biotechnology Co., Ltd. The content of α-ketoglutarate (α-KG) was determined by an kit (BC5420, Beijing Solarbio Science & Technology Co., Ltd.). The contents of isocitric acid (ICA), succinyl coenzyme A (S-CoA), succinic acid (SA), fumaric acid (FA) and oxaloacetic acid (OA) were determined by kits (YX-090301F, YX-190316F, YX-22571F, YX-062113F, YX-150100F, Sino Best Biological Technology Co., Ltd., Beijing, China).

### 2.6 Transcriptome analysis

The mycelia of *F. solani* treated with PGA for 9 h were collected for transcriptome sequencing experiments. The samples were frozen in liquid nitrogen and sent to Shanghai Personal Biotechnology Co., Ltd. for RNA extraction and RNA-Seq sequencing experiments. Criteria for differentially expressed genes (DEGs) were false discovery rate (FDR) < 0.05 and |log_2_FC| > 1. Three biological replicates per treatment. The BioProject accession number for the SRA database: PRJNA1075338.

### 2.7 RT-qPCR verification of differential genes

To verify the reliability of transcriptome data and the expression of TCA-related genes, we screened 6 DEGs related to the TCA cycle for RT-qPCR verification. The primers of RT-qPCR were designed using primer 3.0 software (Supplementary Table S1), and the primers were synthesized by Shanghai Personal Biotechnology Co., Ltd.

The total RNA of mycelium was extracted by the TRIzol method. The quality of the extracted RNA was tested to detect the integrity of 28S and 18S. The main band was clear, single, and bright, and the RNA was good. Total RNA that was tested and quantified was reverse transcribed into cDNA (PrimeScript TM 1st stand cDNA Synthesis Kit). Then, a fluorescence quantitative PCR and a real-time PCR reaction were carried out. The RT-qPCR system was set as follows: 95°C denaturation 5 min, 40 cycles of 95°C for 15 s, followed by 60°C for 30 s, and a dissociation step. The relative gene expression was expressed by normalized DEGs to the internal control gene, Actin, using the 2^-ΔΔCT^ method (Livak and Schmittgen, 2001).

### 2.8 Statistical analysis

Each of the above experiments was repeated three times. Data were expressed as means ± standard errors, and SPSS 26.0 was used for data analysis (*p* < 0.05). Origin 18.0 was used for mapping.

## 3 Results

### 3.1 The inhibitory effect of PGA on *Fusarium solani*

**Figure 2:**
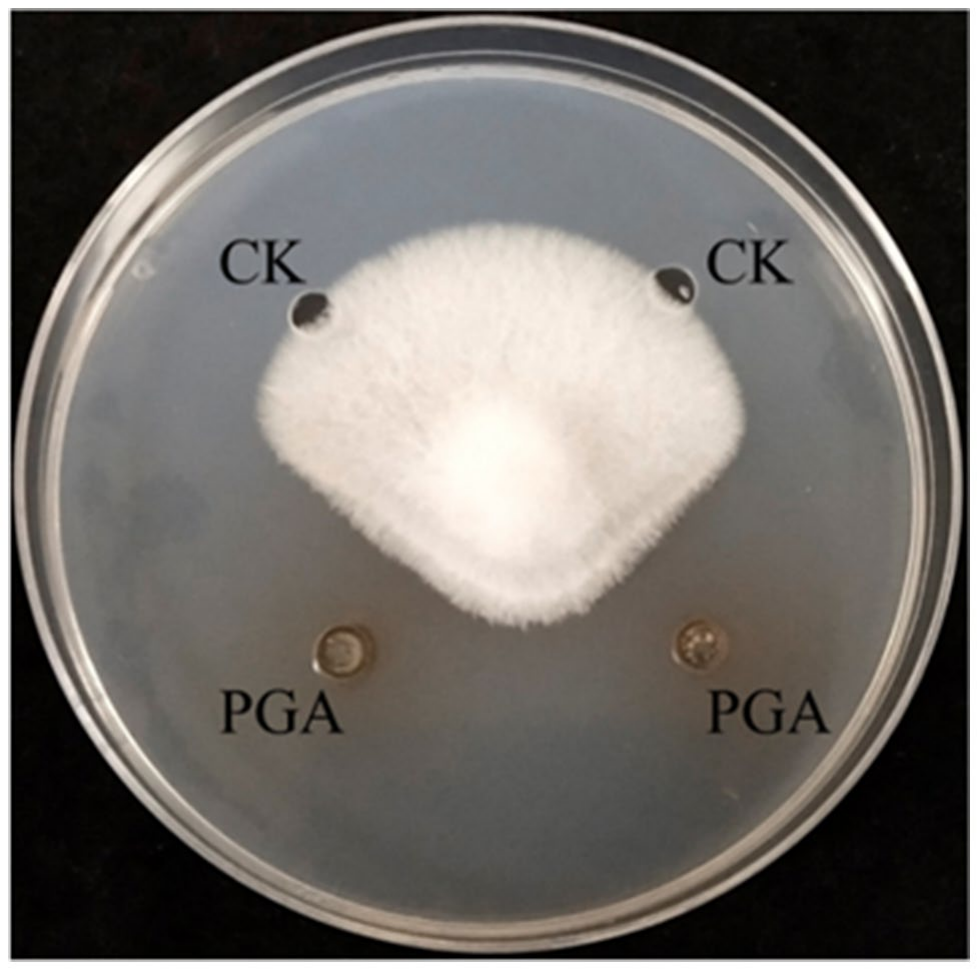
Inhibitory effect of PGA on *Fusarium solani*. PGA: PGA and CK: control (sterile water).

The diameter of PGA inhibiting the growth of *F. solani* was 12.26 mm, and the inhibition rate was 54.49% (Figure 2).

### 3.2 Effect of PGA on mitochondria of *Fusarium solani*

**Figure 3:**
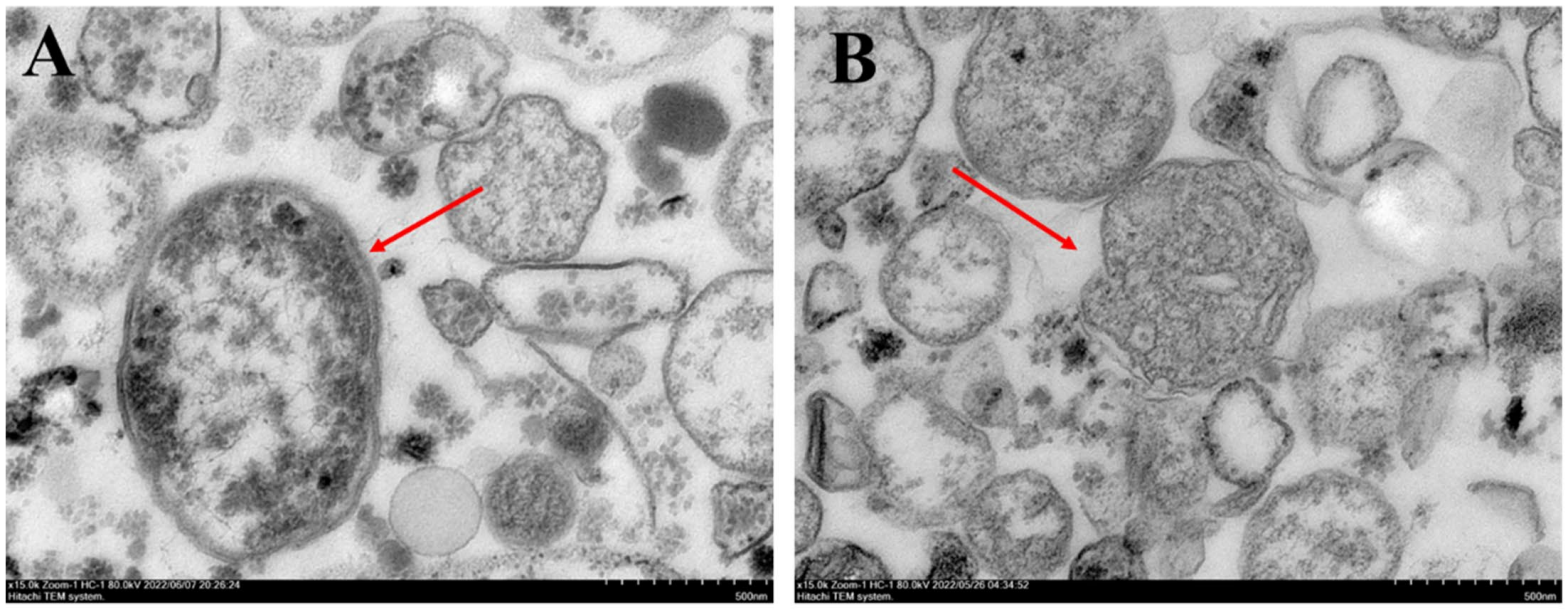
Observation of sitochondrial structure of *Fusarium solani*. **(A)** Control and **(B)** PGA.

The mitochondrial morphology of *F. solani* in the control treatment was primarily round or oval, the internal structure was regular, the surface was smooth and dense, and the mitochondrial inner ridge was complete (Figure 3A). After PGA treatment, the mitochondria of *F. solani* showed a myelin-like layered structure, the membrane tissue ruptured, the mitochondrial inclusions gradually lost, and the mitochondria died (Figure 3B).

### 3.3 Effects of PGA on TCA-related enzyme activities of *Fusarium solani*

**Figure 4:**
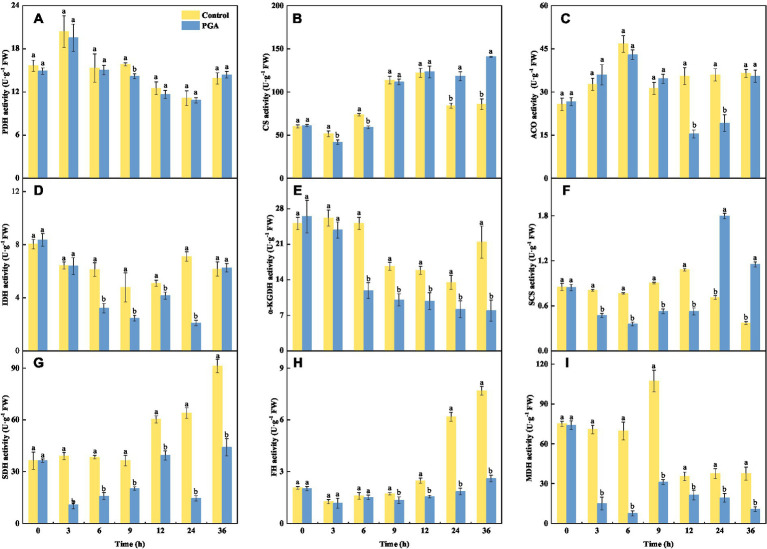
Effects of PGA on the activities of PDH **(A)**, CS **(B)**, ACO **(C)**, IDH **(D)**, α-KGDH **(E)**, SCS **(F)**, SDH **(G)**, FH **(H)** and MDH **(I)** of *Fusarium solani*. Different lowercase letters indicated significant differences between the control and PGA treatments (*p* < 0.05).

At 9 h, the PDH activity of *F. solani* treated with PGA decreased significantly, the activity of PDH was 10.50% lower than the control (Figure 4A; *p* < 0.05). At 12 and 24 h, the ACO activity of PGA-treated *F. solani* was significantly reduced (Figure 4C). During the incubation period, the changes of CS and SCS activities of *F. solani* were consistent, both of which were significantly decreased in the early stage of PGA treatment, and significantly increased in the later stage of treatment (24, 36 h; Figures 4B,F; *p* < 0.05). At 6 and 12 h, the SCS activity of *F. solani* treated with PGA was 53.14 and 51.69% lower than that of the control, respectively. At 24 and 36 h, the activity of CS was 40.70 and 64.15% higher than that of the control, respectively. After PGA treatment, the activities of IDH (Figure 4D), α-KGDH (Figure 4E), SDH (Figure 4G), FH (Figure 4F), and MDH (Figure 4I) of *F. solani* were significantly decreased (*p* < 0.05). After PGA treatment, the MDH activity of *F. solani* treated with PGA was 89.23 and 71% lower than that of the control at 6 and 9 h, respectively. At 24 h, IDH, SDH, and FH activities were lower than those of the control group by 70.44, 77.3, and 70.07%, respectively. At 36 h, the α-KGDH activity of *F. solani* treated with PGA was 62.96% lower than that of the control.

### 3.4 Effect of PGA on TCA organic acid content of *Fusarium solani*

**Figure 5:**
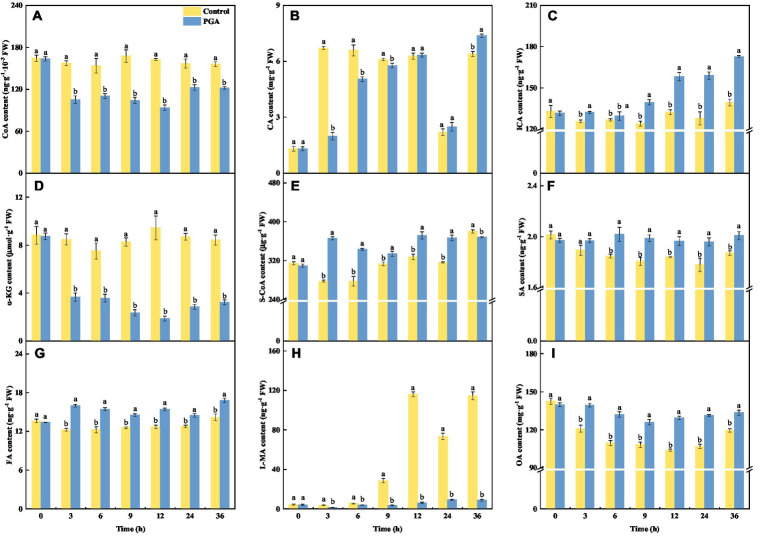
Effects of PGA treatment on the contents of CoA **(A)**, CA **(B)**, ICA **(C)**, α-KG **(D)**, S-CoA **(E)**, SA **(F)**, FA **(G)**, L-MA **(H)** and OA **(I)** of *Fusarium solani*. Different lowercase letters indicated significant differences between the control and PGA treatments (*p* < 0.05).

After PGA treatment, the CA content of *F. solani* decreased first and then increased (Figure 5B). At 3 and 6 h, the CA content of *F. solani* treated with PGA was 73.64% and 23.34% lower than that of the control, respectively. After PGA treatment, the contents of ICA (Figure 5C), S-CoA (Figure 5E), SA (Figure 5F), FA (Figure 5G), and OA (Figure 5I) in *F. solani* increased significantly (*p* < 0.05). At 3 h, the S-CoA and FA contents of *F. solani* treated with PGA were 31.81 and 30.26% higher than those of the control, respectively. At 12 and 24 h, the OA content of *F. solani* treated with PGA was 24.32 and 22.80% higher than that of the control, respectively. At 24 h, the ICA and SA contents of *F. solani* treated with PGA were 24.39 and 10.30% higher than those of the control, respectively. After PGA treatment, the contents of CoA (Figure 5A), α-KG (Figure 5D), and L-MA (Figure 5H) in *F. solani* decreased significantly (*p* < 0.05). At 12 h, the CoA, α-KG, and L-MA of *F. solani* treated with PGA were 42.32, 80.09, and 94.89% lower than those of the control, respectively.

### 3.5 Transcriptome analysis

#### 3.5.1 Quality statistics and analysis of RNA-Seq data

Sterile distilled water control and PGA treatment were set up in the experiment, with 3 biological replicates each, and 6 cDNA libraries were established. After sequencing, the average clean reads of *F. solani* under control and PGA treatment were 63,558,391 and 51,690,066, respectively. Q20 (the proportion of bases with sequencing quality above 99.0% of the total bases) averaged 98.52% and 98.60%, respectively, and Q30 (the proportion of bases with sequencing quality above 99.9% of the total bases) averaged 95.52% and 95.79%, respectively (Supplementary Table S2). The above results indicated that the cDNA library obtained by this sequencing is of high quality and can be further studied by subsequent bioinformatics.

#### 3.5.2 Analysis of DEGs

**Figure 6:**
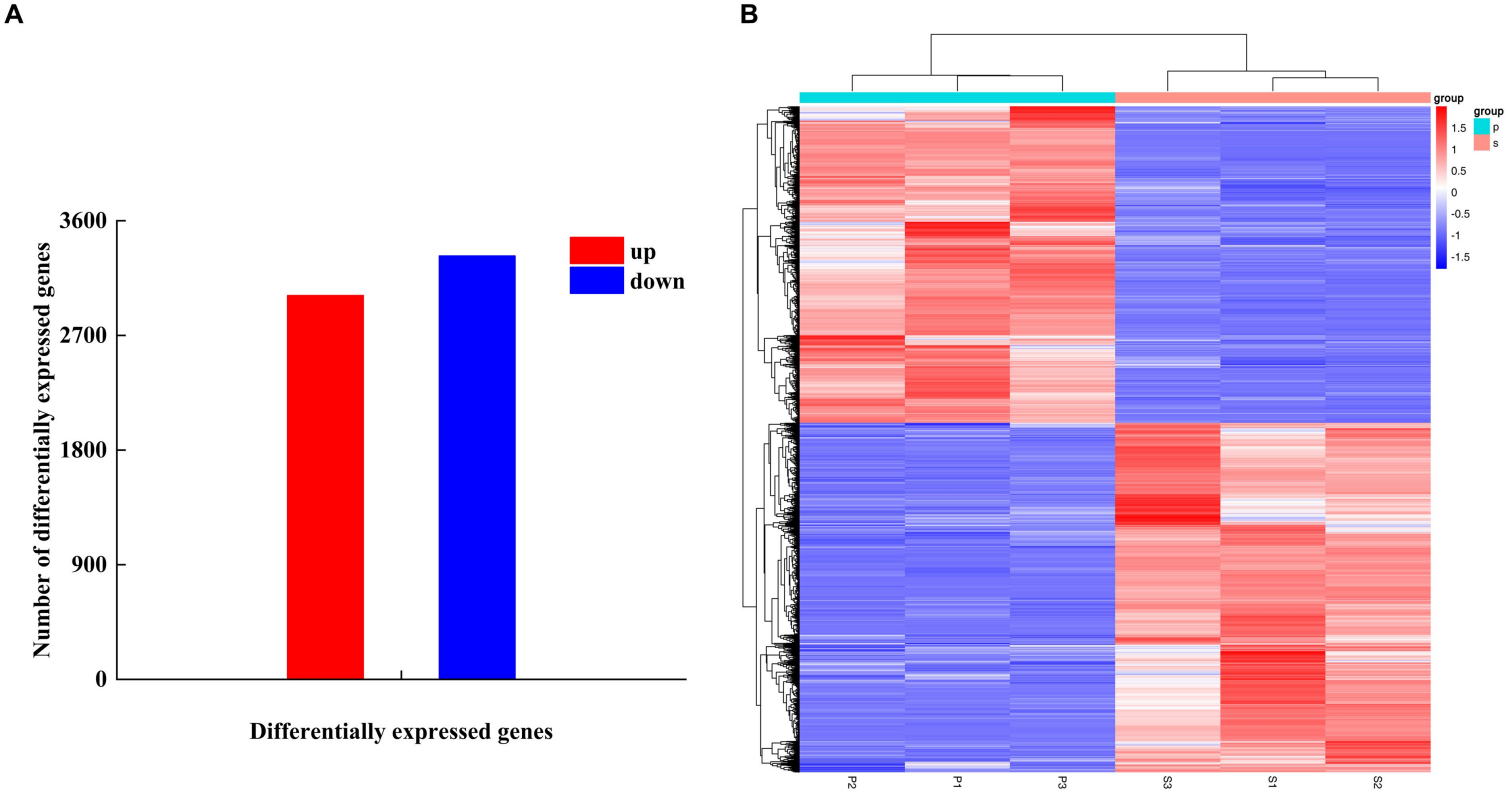
The number of DEGs **(A)** and cluster heat map **(B)** were used to analyze the DEGs of *Fusarium solani* after PGA treatment. S indicates control, P indicates addition of PGA.

After PGA treatment of *F. solani*, a total of 6,341 genes were changed in expression, of which 3,014 genes were up-regulated and 3,327 genes were down-regulated (Figure 6A). Clustering heatmap analysis showed that there were significant differences in gene expression profiles between the treatment and the control (Figure 6B). Six DEGs related to TCA cycle were screened for RT-qPCR verification.

#### 3.5.3 GO analysis of DEGs

**Figure 7:**
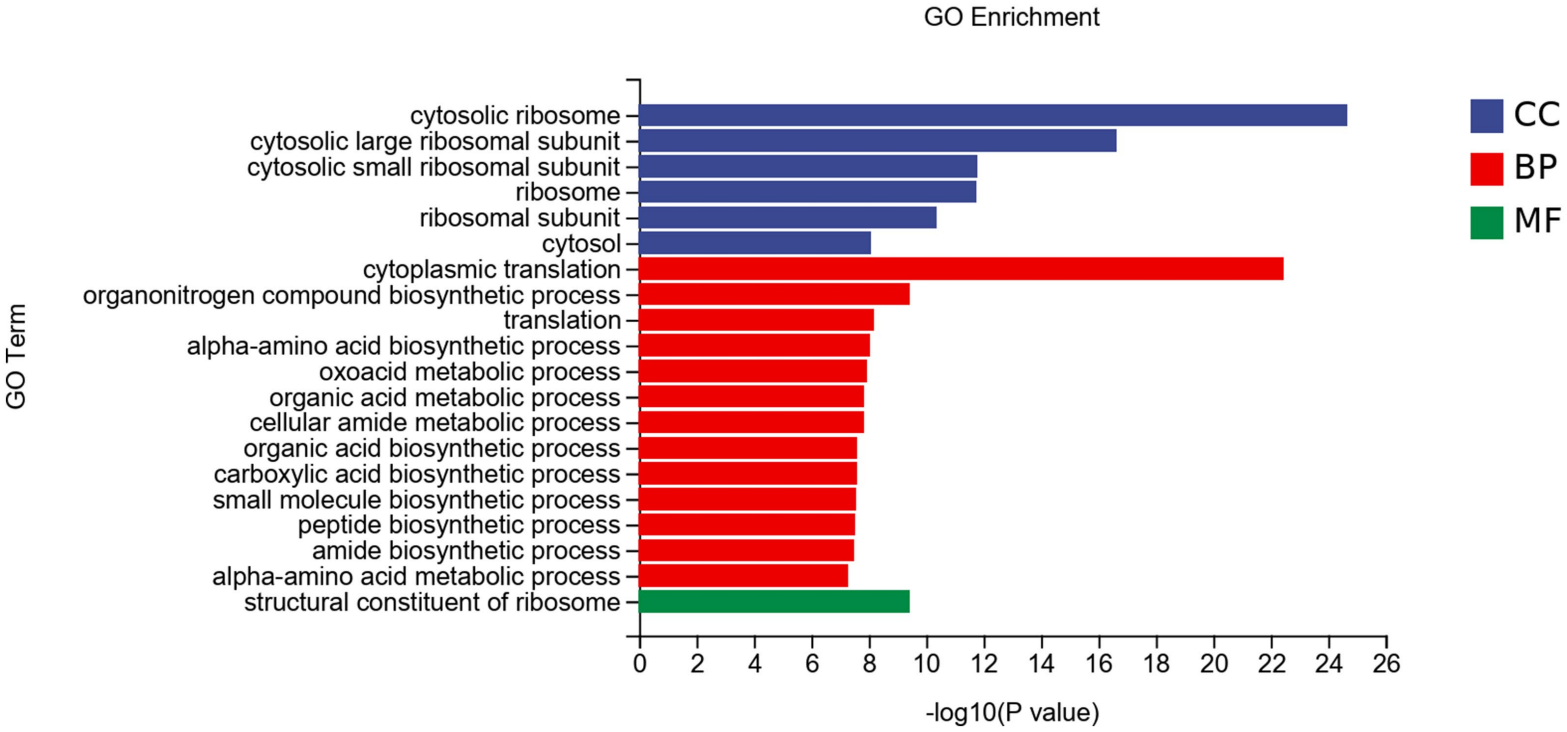
GO enrichment analysis of DEGs of *Fusarium solani* after PGA treatment. CC, Cellular component; BP, Biological Process; MF, Molecular function.

The DEGs were divided into three categories: biological processes (BP), cellular components (CC), and molecular functions (MF; Figure 7). In CC, DEGs were mainly enriched in the cytosolic ribosome et al. In BP, DEGs were mainly enriched in cytoplasmic translation et al. DEGs enriched in MF were less, mainly enriched in the structural constituent of ribosome.

#### 3.5.4 KEGG analysis of DEGs

**Figure 8:**
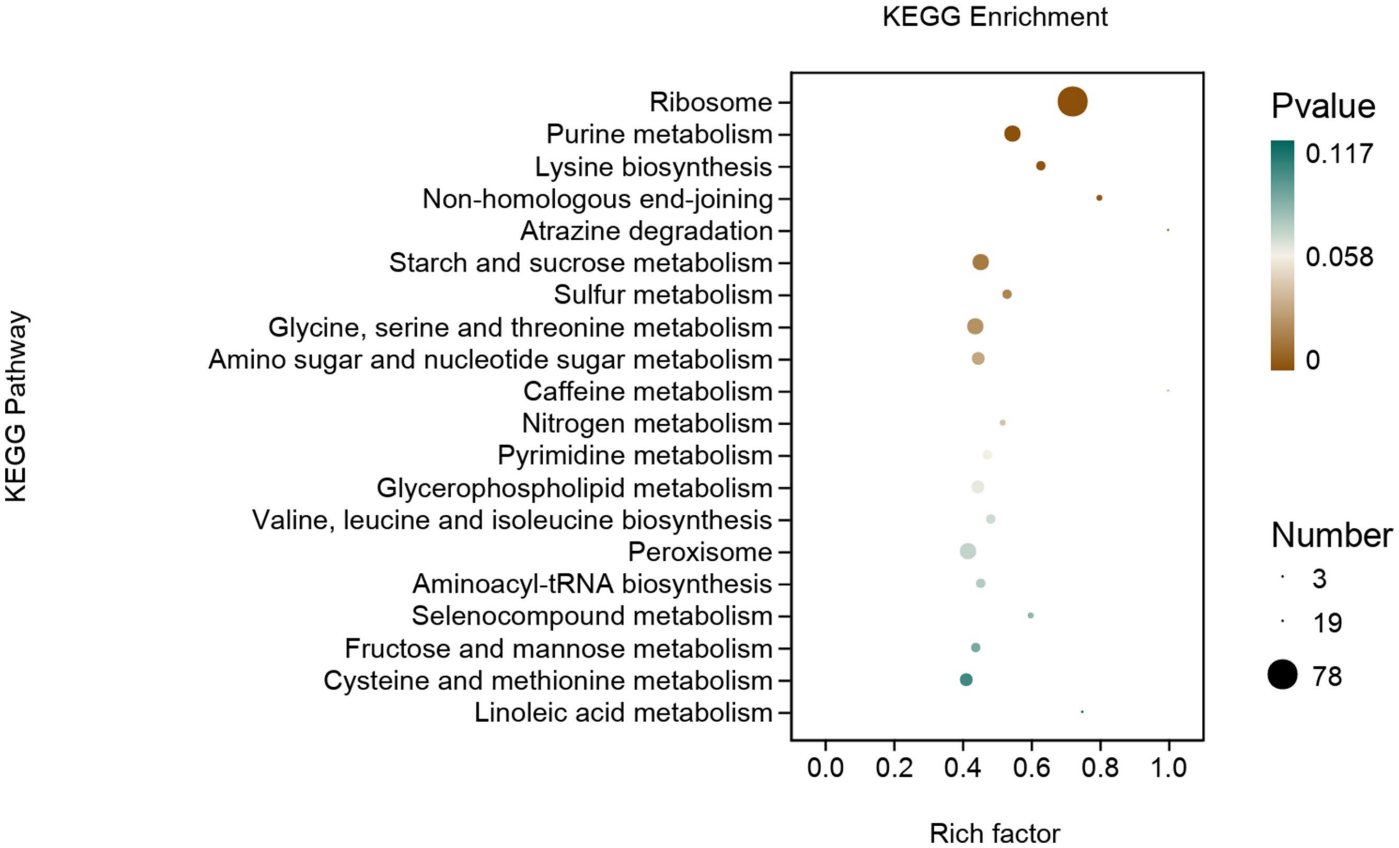
KEGG enrichment analysis of DEGs of *Fusarium solani* after PGA treatment.

DEGs were mainly enriched in genetic information processing, metabolism, and cellular processes (Figure 8). In genetic information processing, DEGs were mainly enriched in ribosomes, non-homologous end-joining, and aminoacyl-tRNA biosynthesis. In metabolism, DEGs were mainly enriched in purine metabolism, lysine biosynthesis et al. The DEGs of cellular processes are mainly enriched in peroxisomes.

### 3.6 The effect of PGA on the TCA cycle of *Fusarium solani* was determined by transcriptomics

**Figure 9:**
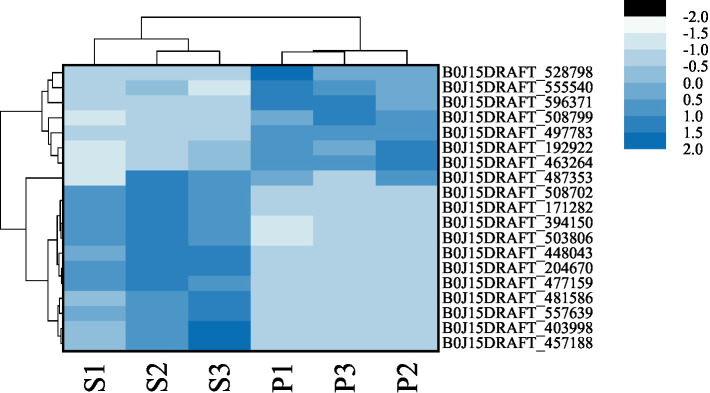
The effect of PGA on the TCA cycle of *Fusarium solani* was determined by transcriptomics. S indicates control, and P indicates addition of PGA.

We evaluated whether the expression levels of TCA cycle-related genes in the mycelium of *F. solani* changed after PGA. Searching according to the KGEE database. After PGA treatment, the expression levels of 19 genes were significantly changed, resulting in changes in the corresponding enzyme activities, indicating that PGA could significantly affect the normal operation of the TCA cycle of *F. solani* (Figure 9).

### 3.7 RT-qPCR validation of DEGs

**Figure 10:**
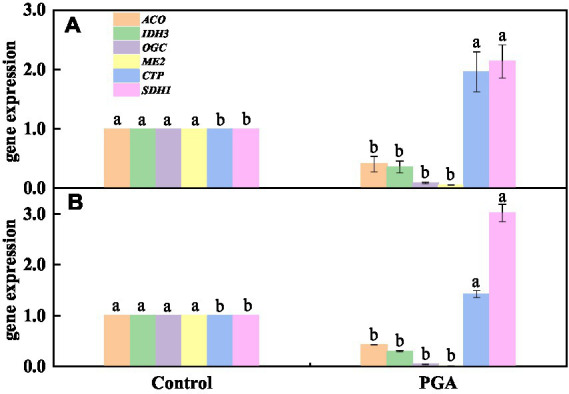
Verification results of DEGs. **(A)** Transcriptome results; **(B)** RT-qPCR.

The results showed that the verification results of 6 DEGs related to the TCA cycle were consistent with transcriptome sequencing, but there was a specific deviation in gene expression multiples (Figures 10A,B). After PGA treatment, 4 TCA-related candidate genes (ACO, IDH3, OGC, ME2) were significantly down-regulated (Figure 10B). Two TCA-related candidate genes (CTP, SDH1) were significantly up-regulated (Figure 10B).

## 4 Discussion

In this study, the effects of PGA on colony growth, mitochondrial ultrastructure, main enzyme activities and product content of the TCA cycle in *F. solani* were comprehensively analyzed. Transcriptomics technology was used to analyze the differences in mycelium at the transcriptional level.

The diameter of the colony is of great significance to the growth of pathogenic fungi (Luo and Tian, 2021). PGA could inhibit the colony growth of pathogenic fungi such as *A. solani* (Wolters et al., 2023) and *P. brasiliense* (Joshi et al., 2021). PGA inhibited the mycelial growth rate and spore germination of *C. trifolii* (Xu et al., 2023), and inhibited the growth of *F. sulphureum* by inhibiting ROS metabolism and destroying cell membrane (Li et al., 2023).

It has been reported that the ultrastructural changes in mitochondria may be due to the leakage of small molecules and the damage of mitochondria caused by the difference in cell metabolism (Bajpai et al., 2013). In this study, after PGA treatment, the mitochondrial membrane tissue of *F. solani* was broken, and the contents were lost. This was similar to the results of the mitochondria of *B. cinerea* treated with tea tree oil (Li et al., 2017) and the mitochondria of *Saccharomyce scerevisiae* treated with essential oil from *Chrysanthemum morifolium* cv. Fubaiju (Zhan et al., 2021). It indicated that PGA treatment changed the mitochondrial ultrastructure of *F. solani*, which may affect the ability of mitochondria to metabolize.

One of the central pathways of metabolism is the TCA cycle, and it is also the primary mechanism of energy production. Mitochondrial dehydrogenase is an essential enzyme in biological growth. The decrease of a-KGDH activity leads to a decrease in the ability of TCA to produce NADH and ATP synthesis. It leads to the disorder of the TCA cycle (Huang et al., 2003). By linking the utilization of nutrients with the synthesis of TCA cycle intermediates and products, CS helps regulate energy flux and metabolic rate. It catalyzes the condensation of acetyl coenzyme A and oxaloacetic acid to produce citric acid, coenzyme A, and proton (Liao et al., 2014). The activity of IDH regulates the flow of isocitrate into the TCA cycle or glyoxylic acid cycle (Li et al., 2017). The decrease in MDH activity inhibited the conversion between malic acid and oxaloacetic acid (Kobayashi et al., 2002). SDH can transfer two electrons in the electron transport chain to coenzyme Q10, the intersection of the TCA cycle and oxidative phosphorylation. Decreased activity can affect the regular operation of oxidative phosphorylation and electron transport and is also a marker protein for evaluating mitochondrial function (Evenepoel et al., 2015; Zou et al., 2015). In this study, PGA could inhibit the respiratory pathway of *F. solani* by reducing the activities of ACO, IDH, α-KGDH, SCS, SDH, FH, and MDH. *Cinnamon oil* inhibited the expected growth of Rhizopus nigricans by reducing SDH and MDH activities (Fan et al., 2022). O-vanillin treatment inhibited the activities of CS, IDH, a-KGDH, and SDH of *A. flavus*, thereby reducing its virulence (Li et al., 2022). Citral decreased the activities of IDH, a-KGDH, and SDH in *P. digitatum* and inhibited its normal metabolism (Zheng et al., 2015). Eugenol and citral reduced the activities of MDH, CS, α-KGDH, IDH, and SDH in *A. niger* (Ju et al., 2023). The above results are consistent with the results of this experiment. This indicated that the mitochondrial function of pathogenic fungi was significantly inhibited.

The TCA cycle converts phosphoenolpyruvate to malate and/or pyruvate in the cytosol. These organic acids are then taken into the mitochondria, producing energy (Fernie et al., 2004). After PGA treatment, the content of α-KG and L-MA in *F. solani* decreased significantly, indicating that the TCA cycle was significantly inhibited. The reason for this inhibition may be that PGA inhibits the activity of some amino acids in *F. solani*, and the TCA cycle is the final stage of these amino acid metabolisms. Therefore, some metabolites in the TCA cycle could be reduced (Gaupp et al., 2010). The decrease of TCA cycle flux further leads to the decrease of CoA synthesis. The decrease of IDH, SCS, SDH, FH, and MDH activities led to accumulating the corresponding reaction substrates ICA, S-CoA, SA, FA, and OA, further blocking the TCA cycle and causing mitochondrial dysfunction. It has been reported that Graphene oxide can significantly inhibit the mycelial growth of *F. graminearum*, and the content of SA and CA increased significantly (Wang et al., 2019). This is consistent with the results of this experiment. Correlation analysis showed a significant negative correlation between α-KGDH and S-CoA, and a positive correlation between α-KGDH and α-KG. The results showed that the content of α-KG and the activity of α-KGDH decreased, but the content of S-CoA increased (Supplementary Table S3). It may be due to the decrease in SCS activity, resulting in S-CoA cannot be consumed in time. There was a significant positive correlation between L-MA and FH, α-KG and IDH (Supplementary Table S3), indicating that the corresponding product content decreased with the decrease of enzyme activity. CS was significantly negatively correlated with OA, and CS was significantly positively correlated with *CA.* That is the content of substrate OA increased, the activity of CS decreased, and the content of product CA decreased.

In this experiment, it was found that after PGA treatment of *F. solani*, GO analysis showed that DEGs were mainly enriched in cell biological processes and components of cells, and the pathway was mainly enriched in genetic information processing and metabolism, indicating that PGA could significantly affect the cell components and metabolism of *F. solani*. The TCA cycle is the hub of sugar, lipid, protein and nucleic acid metabolism communication and transformation (Xu and Shao, 2017). Transcriptome analysis showed that PGA could affect the metabolic pathways of *F. saloni*, such as starch and sucrose metabolism, amino sugar and nucleotide sugar metabolism, fructose and mannose metabolism, and then affect the normal metabolic process of TCA cycle. The up-regulation of SDH-related gene (*SDH1*) expression leads to defects in enzyme structure and function, and the resulting electron leakage and cell damage lead to an increase in reactive oxygen species (ROS). ROS concentration in mitochondria increases DNA damage, disrupting normal cell metabolism (Baysal et al., 2000). Decreased expression of IDH-related genes can inhibit the production of α-KG, resulting in abnormal expression of signal transduction pathways (Chou et al., 2010). FH is involved in the TCA cycle in mitochondria and catalyzes the conversion of fumaric acid to malic acid. In this experiment, due to the addition of PGA, the expression level of the FH-related gene (*CTP*) was up-regulated, resulting in a decrease in its activity, which in turn led to a decrease in malic acid content, which was consistent with the results of Ju et al. (2023). The decreased expression levels of α-KGDH, ACO, and MDH-related genes (*OGC*, *ACO*, *ME2*) led to a significant change in their activity, further leading to a decrease in the traffic of the TCA cycle pathway.

## 5 Conclusion

PGA can destroy the mitochondrial ultrastructure of mycelium, regulate the enzyme activity of the TCA cycle, the content of intermediate products, and the expression level of essential genes, cause the disorder of energy metabolism of *F. solani*, and finally inhibit the average growth of *F. solani* to achieve antifungal effect.

## Data availability statement

The datasets presented in this study can be found in the NCBI BioProject Database (https://www.ncbi.nlm.nih.gov/bioproject), accession number PRJNA1075338.

## Author contributions

CZ: Conceptualization, Data curation, Methodology, Software, Supervision, Writing – original draft, Writing – review & editing. WC: Writing – original draft, Conceptualization. BW: Conceptualization, Supervision, Writing – original draft. YW: Writing – original draft, Methodology, Conceptualization. NL: Writing – original draft, Software, Methodology. RL: Writing – original draft. YY: Methodology, Writing – original draft. YS: Methodology, Writing – original draft. JH: Writing – review & editing, Writing – original draft, Supervision, Methodology, Funding acquisition, Data curation, Conceptualization.
