# Supplementary material for: Potato glycoside alkaloids exhibit antifungal activity by regulating the tricarboxylic acid cycle pathway of Fusarium solani
Source: Front Microbiol. 2024 Apr 15;15:1390269. doi: 10.3389/fmicb.2024.1390269 (PMC11056507; doi:10.3389/fmicb.2024.1390269)
Supplement: Supplementary file 1 [file Data_Sheet_1.docx]

# Potato glycoside alkaloids exhibit antifungal activity by regulating the tricarboxylic acid cycle pathway of *Fusarium solani*

Chongqing Zhang^1^, Wei Chen^1^, Bin Wang^1^, Yupeng Wang^1^, Nan Li^1^, Ruiyun Li^1^, Yuke Yan^1^, Yuyan Sun^1^, and Jing He^1,2*^

^1^ College of Forestry, Gansu Agricultural University, Lanzhou 730070, China;

^2^ Wolfberry Harmless Cultivation Engineering Research Center of Gansu Province, Lanzhou 730070, China

*** Correspondence:**Jing He
hejing268@aliyun.com

# Supplementary Tables

Table. S1. Primer sequences

| gene | Accession no. | forward primer | reverse primer |
| --- | --- | --- | --- |
| *ACO* | B0J15DRAFT_403998 | 5’-TACAACTTCCTTGCTACT-3’ | 5’-GAGAATAATCTGGTGAATGA-3’ |
| *IDH3* | B0J15DRAFT_457188 | 5’-AGGTCAACGACATGATTG-3’ | 5’-CAGGATACCACCGTAAAG-3’ |
| *OGC* | B0J15DRAFT_204670 | 5’-GATTCTACCGTGGCTTCG-3’ | 5’-TAGTCAGCAACGATCAGTG-3’ |
| *SDH1* | B0J15DRAFT_508799 | 5’-GATATGCCTAAGAAGAATAAG-3’ | 5’-TGATACTGAAGAGATGGA-3’ |
| *ME2* | B0J15DRAFT_477159 | 5’-TCACTCAAGGACCAGAAT-3’ | 5’-AACAACGCTCATCATCTC-3’ |
| *CTP* | B0J15DRAFT_192922 | 5’-GTCACCTTCACCGTCTAC-3’ | 5’-CGTACTTGCCTCCAACAA-3’ |

Table. S2. Statistics of sequencing data quality

| Sample | Clean Reads | Raw Q20 rate(%) | Raw Q30 rate(%) |
| --- | --- | --- | --- |
| S1 | 52327538 | 98.6 | 95.72 |
| S2 | 74178468 | 98.52 | 95.55 |
| S3 | 64169168 | 98.45 | 95.29 |
| P1 | 44760642 | 98.6 | 95.81 |
| P2 | 52225998 | 98.65 | 95.95 |
| P3 | 58083558 | 98.54 | 95.62 |

Note: S indicates control, P indicates addition of PGA.

Table. S3. The correlation between the main enzyme activities of TCA cycle and the content of organic acids

|  | SCS | MDH | α-KGDH | CS | FH | SDH | IDH | PDH | ACO |
| --- | --- | --- | --- | --- | --- | --- | --- | --- | --- |
| SA | -0.209 | -0.237 | -0.199 | -0.001 | 0.053 | 0.014 | -0.042 | 0.110 | .534* |
| OA | 0.001 | 0.370 | .771** | -.638** | 0.129 | 0.017 | .802** | .569** | 0.140 |
| S-CoA | 0.235 | -.783** | -.493* | .434* | 0.014 | -0.021 | -0.307 | -0.145 | -0.227 |
| CA | -0.242 | -.499* | -.722** | .662** | 0.182 | .496* | -0.326 | -0.349 | 0.022 |
| L-MA | .812** | -0.197 | -.648** | .803** | .715** | 0.411 | -0.241 | -.724** | -0.432 |
| FA | -0.134 | -.795** | -0.358 | 0.246 | 0.180 | 0.132 | 0.006 | 0.253 | 0.336 |
| α-KG | 0.020 | .823** | .755** | -.523* | 0.251 | 0.193 | .732** | 0.266 | 0.057 |
| CoA | 0.370 | .765** | .511* | -0.225 | .504* | 0.263 | .588** | 0.016 | -0.043 |
| ICA | .605** | -0.349 | -.658** | .898** | .621** | .538* | -0.149 | -.562** | -0.426 |

Note: * *P*<0.05; ** *P*<0.01.
